# Diet and microbiome shape small-molecule cytokinin pools in mammals

**DOI:** 10.1080/19490976.2026.2679497

**Published:** 2026-06-12

**Authors:** Eman M. Othman, Elena Bencurova, Pamela Ferretti, Peer Bork, Alvaro Rodriguez del Rio, Jaime Huerta-Cepas, Ignazio Caruana, Rania Abdel-latif, Aman Akash, Alfonso Albacete, Feras Lafi, Thomas Dandekar, Muhammad Naseem

**Affiliations:** a Department of Biochemistry, Faculty of Pharmacy, Minia University, Minia, Egypt; b Julius-Maximilians-Universität Würzburg, Department of Bioinformatics, Theodor-Boveri-Institute, Biocenter, Würzburg, Germany; c Julius-Maximilians-Universität Würzburg, Department of Biochemistry-I, Theodor-Boveri-Institute, Biocenter, Würzburg, Germany; d European Molecular Biology Laboratory, Structural and Computational Biology Unit, Heidelberg, Germany; e Section of Genetic Medicine, Department of Medicine, University of Chicago, Chicago, IL, USA; f Centro de Biotecnología y Genómica de Plantas, Universidad Politécnica de Madrid (UPM) – Instituto Nacional de Investigación y Tecnología Agraria y Alimentaria (INIA-CSIC), Madrid, Spain; g Department of Pediatrics – Hematology, Oncology and Stem Cell Transplantation Unit, University Hospital Würzburg, Würzburg, Germany; h Department of Pharmacology and Toxicology, Faculty of Pharmacy, Minia University, Minia, Egypt; i Instituto Murciano de Investigación y Desarrollo Agrario y Alimentario, Murcia, Spain; j College of Natural and Health Sciences, Department of Environmental Science and Sustainability, Zayed University, Abu Dhabi, United Arab Emirates

**Keywords:** Cytokinins, diet-gut nexus, plant hormone, kinetin, t-zeatin

## Abstract

Cytokinins (CKs) are adenine-derived metabolites traditionally characterized as plant hormones, yet their origin, distribution, and functions in mammalian systems remain largely undefined. Using integrated metabolomics, microbiome, and metagenomics approaches, we provide a systematic characterization of CK occurrence and potential sources in mammals. Serum profiling across five animal species revealed consistent detection of multiple CK derivatives, with concentrations markedly lower than in plant tissue. The CK storage form, zeatin-O-glucoside, predominated in mammalian sera, followed by trans-zeatin and kinetin, indicating a CK composition distinct from that in plants. Species-specific differences, such as reduced trans-zeatin in mice and lower kinetin in humans, further suggest divergent regulatory patterns. In mice, CKs were present in vascular tissues of the kidney, heart, and liver, demonstrating systemic distribution. Dietary manipulation showed that starvation significantly reduced CK abundance in serum, colon, feces, and urine, confirming that diet is a major contributor to the mammalian CK pool. Meta-omics analysis of gut microbiomes identified CK-related genes across multiple microbial taxa, with the highest representation in human microbiomes, followed by those of mouse and pig. Germ-free mouse experiments showed substantially lower CK levels than conventionally raised counterparts, establishing a microbiome-dependent contribution. Collectively, our findings identify CKs as diet and microbiome modulated metabolites in mammals, warranting future investigation to elucidate their physiological significance in mammalian biology.

## Introduction

Cytokinins (CKs), adenine-derived small-molecule hormones, are key regulators of plant growth, development, and stress responses. They are categorized by their N6-side chains, which determine their biological activity. Isoprenoid CKs such as N6-isopentenyladenine (iP), trans-zeatin (tZ), cis-zeatin (cZ), and dihydrozeatin (DZ) are the most common and naturally occurring forms.^1^ Despite their widespread presence across various forms of life,^1^ ^,^ ^2^ the endogenous roles of CKs have not been systematically studied beyond the kingdom Plantae. The exogenous application of CKs holds strong therapeutic potential, including their ability to mitigate oxidative stress,^3^ ameliorate various pathophysiological conditions,^4^ and attenuate viral replication and inflammation.^5^ They show promise in neurodegenerative diseases such as Huntington's disease (HD)^6^ ^,^ ^7^ and Familial Dysautonomia (FD),^8^ and have potential as potent cosmeceuticals.^9^ CKs also contribute to muscle growth,^10^ ^,^ ^11^ anti-senescence processes,^12^ and healthy ageing and longevity.^13‐15^ Despite these promising translational benefits, the detailed endogenous physiological roles of CKs in mammalian systems remain to be elucidated. Therefore, a comprehensive investigation into the origins and mechanisms of endogenous CK production is essential for a better understanding of its biological significance in mammalian systems.

In this regard, tRNA-isopentenyltransferase 1 (TRIT1; EC 2.5.1.75; sketched placement in domain types and phylogeny are shown in Supplementary material, Figure 1), an enzyme that modifies the A37 position of mitochondrial and cytosolic tRNAs, has been previously associated with the synthesis of iP-type CKs in mammalian systems.^1^ ^,^ ^2^ ^,^ ^10^ However, the pool of endogenous CKs detected in cell cultures and canine samples^1^ ^,^ ^10^ is not limited to this single CK type, and TRIT1 alone cannot suffice for the full spectrum of various CK derivatives. We investigated, for the first time, the gut-food axis as a contributor to CKs in mammalian sera by integrating metabolomics with microbial and metagenomics analysis. Overall, our work uncovers an entirely new line of inquiry into the potential role of the plant hormone CK in mammalian physiology.

**Figure 1.**
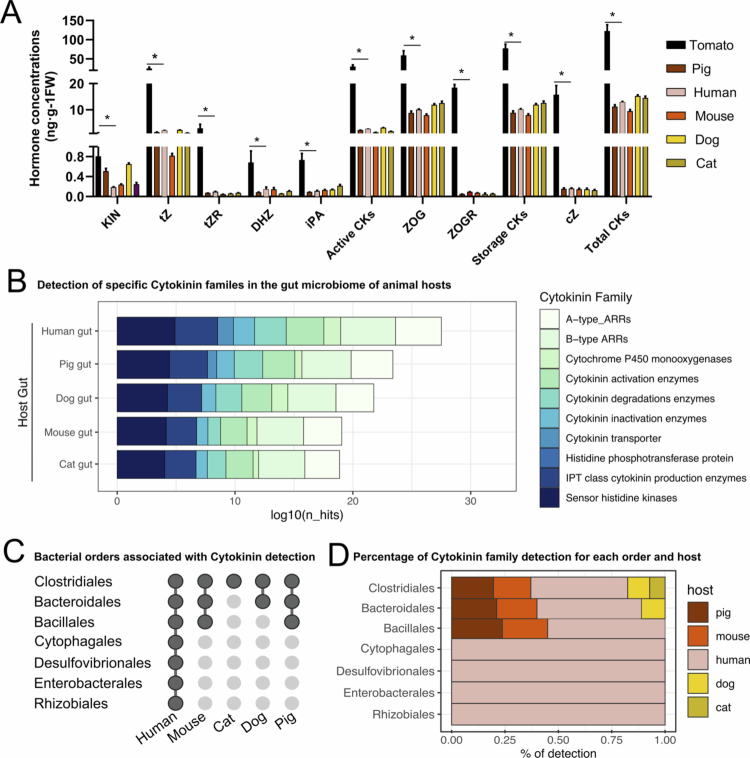
The presence of cytokinin metabolites in mammalian serum samples and metagenomic analysis of their gut microbial communities. Cytokinin hormone concentrations in tomato leaves and mammalian tissues (A), where KIN; kinetin; tZ; trans-zeatin; iZR; isopentenyladenosine riboside; DHZ; dihydrozeatin; iPA; isopentenyladenine; active CKs; active cytokinins; ZOG; zeatin-O-glucoside; ZOGR; zeatin-O-glucoside riboside; Storage CKs; conjugated storage cytokinins form, cZ; cis-zeatin, total CKs; sum of all quantified cytokinin metabolites. Data are presented as mean ± SEM (*n* = 4–5). Normality was assessed prior to analysis; due to deviation from normal distribution, statistical comparisons were performed using non-parametric methods *p < 0.05 indicates statistically significant differences of cytokinin hormones in tomato leaves compared with mammalian tissues; Mammalian guts in which cytokinin families (IPT, CKX, LOG, AHKs, AHPs, A-type ARRs, B-type ARRs; see colour bar right in (B)) were detected when interrogating the GMGC catalogue (B). All results were based on a minimum e-value threshold of 10^−3^. Microbial taxa at the order level associated with the detection of cytokinin families across animal gut microbiomes (C). Relative percentage of detection of cytokinin families across hosts and microbial orders (D).

A variety of CK derivatives are found in nature; their biological functions are largely determined by the structural variations present in their N6-side chains. To reflect on the biochemical diversity that CKs possess in mammalian sera, we provide the first comprehensive profiling of CKs in serum samples from multiple animal sources (pigs, dogs, mice, cats, and humans), with tomato leaves serving as a positive control (Figure 1A). Animals for serum sampling were selected based on their dietary preferences, ensuring the inclusion of carnivores, herbivores, and omnivores for comparative CK profiling. The levels of iP, tZ, isopentenyladenosine riboside (iZR), cZ, DZ, zeatin-O-glucoside (ZOG), and zeatin-O-glucoside riboside (ZOGR) in plant samples are 4–10 times higher than the corresponding CK levels detected in animal sera, including those from humans. In contrast, the difference between the levels of kinetin in plant and animal samples is relatively modest (Figure 1A). Among all the detected CK types, ZOG, an inactive storage form, was present at higher levels in animal sera compared to other CKs, followed by active forms such as trans-zeatin (tZ) and then kinetin (Figure 1A). These data suggest that CKs may play overlapping but also specialized roles in mammals, distinct from those in plants. Despite variation in the natural diets of the mammalian cohort, overall CK levels were not significantly different among species, although tZ was lower in mice and kinetin was lower in humans. This suggests that CKs may exhibit different species-specific tropisms (Figure 1A).

To gain functional insights into the origin of CKs in the tested mammalian systems, we focused on the mouse model (C57BL/6) and conducted a detailed analysis of various tissues. We detected CKs (ng g^−1^) in the vascular tissues of the kidneys (total CKs: 35.217 ± 1.672; storage CKs: 31.442 ± 2.018), as well as in the heart (total CKs: 35.806 ± 4.273; storage CKs: 33.975 ± 4.226) and liver (total CKs: 30.626 ± 4.833; storage CKs: 27.456 ± 4.165), indicating a systemic distribution in these organs. Notably, no significant differences in CK concentrations were detected among these tissues. However, CK levels in some of these tissues differ significantly from those observed in the mouse serum samples (total CK: 30.577 ± 2.722; storage: 28.690 ± 2.540). These results indicate that CKs are systemically distributed across multiple mouse tissues rather than being confined to a single organ. Similar CK concentrations in the kidney, heart, and liver suggest a common regulatory mechanism or widespread uptake and maintenance in peripheral tissues. In contrast, the significant differences between tissue and serum CK levels suggest that circulating CKs may not directly reflect tissue-specific CK pools, highlighting the potential for localized regulation or metabolism within organs.

To explore how the diet–gut microbiome axis influences circulating CK levels, we conducted meta-omics analyses on the gut microbiomes of the same animals in which serum CK concentrations were assessed (Figure 1A). We identified CK-related pathway genes (Table 1) in the Global Microbial Gene Catalogue (GMGC) (https://gmgc.embl.de/), a comprehensive metagenomic database encompassing over 2.3 billion ORFs from 13,174 metagenomic samples, spanning 14 habitats.^16^ Most of these mammalian guts harbored microbiomes enriched for genes related to CK signaling and metabolic pathways (Table 1). The maximum hits received by each taxon are in the human gut, followed by mouse and pig, while the guts of cats and dogs received relatively fewer hits than other animal species (Figure 1B). CK-specific genes were identified across microbial orders, including Clostridiales, Bacteroidales, Bacillales, Cytophagales, Desulfovibrionales, Enterobacterales, and Rhizobiales (Figure 1C and D), with Clostridiales common to all the studied animals. Notably, only in human samples could CK-specific sequences be detected in Cytophagales, Desulfovibrionales, Enterobacterales, and Rhizobiales (Figure 1C and D). These orders are not exclusive to humans, but they were absent from the mouse samples. Additional factors that may influence the microbiome include dietary structure, the composition of the gut microbiota, sequencing depth, database bias, and other variables.

**Table 1.** Overview on cytokinin-related proteins^a^.

Gene (full name)	Function	AGI & citation
Isopentenyltransferase (IPT)	First step in cytokinin biosynthesis	At1g68460.1; Miyawaki et al., 2004, *Plant Cell* 16:2665
Lonely Guy (LOG) Cytokinin-Activating Enzyme	Converts nucleotide cytokinins to active free bases	At2g28305.1; Kurakawa et al., 2007, *PNAS* 104:5001
UDP-Glucosyltransferase	Conjugates cytokinins to sugars (inactivation)	At5g05870.1; Hou et al., 2004, *Plant Physiol* 134:1732
Cytokinin Oxidase/Dehydrogenase (CKX)	Irreversible cytokinin degradation	At2g41510.1; Schmülling et al., 2003, *Nature* 421:608
Cytochrome P450 CYP735A	Hydroxylation of iP-type cytokinins to trans-zeatin	At5g38450.1; Takei et al., 2004, *PNAS* 101:8821
Purine/Cytokinin Transporter	Transport of cytokinins across membranes	At1g19770.1; Bürkle et al., 2003, *Plant Physiol* 131:278
Arabidopsis Histidine Kinase Receptor (AHK)	Cytokinin receptor initiates phosphorelay	At2g17820.1; Inoue et al., 2001, *Nature* 409:1060
Histidine Phosphotransfer Protein (AHP)	Transfers phosphate to response regulators	At1g03430.1; Suzuki et al., 2001, *Plant Cell* 13:1155
B-Type Arabidopsis Response Regulator (ARR1)	Transcription factor activating cytokinin-responsive genes	At3g16857.1; Sakai et al., 2001, *Plant Cell* 13:703
A-Type Arabidopsis Response Regulator (ARR4)	Negative feedback regulator of cytokinin signaling	At1g59940.1; To et al., 2004, *Plant Cell* 16:658

^a^ Cytokinin-related proteins, grouped by function, along with their Arabidopsis gene IDs (“AGI”), functions, and notable features, were queried into the Global Microbial Gene Catalogue (GMGC) database to detect CK-related gene families in microbial lineages of mammalian gut inhabitants. Supplementary material document file provides methods details, the Supplementary Excel Tables 1–4 provide details for these candidates (A- and B-type response regulator Tables 1 and 2; Lonely guy activating enzyme Table 3; degradation enzyme CKX Table 4) with domain and protein family annotations (PFAM, SMART, eggNOG) from the GMGC database.

However, these data support the idea that gut microbiota can contribute to CK biosynthesis across taxa. While CK-related cellular circuitry is well characterized only in plants,^17^ ^,^ ^18^ knowledge of the genetic basis of CK signaling and metabolism in microbial systems is still emerging.^19‐21^ Our metagenomic analysis suggests that while complete CK signaling and biosynthesis pathways may not be fully present within all microbial taxa, their components are distributed and collectively represented across diverse taxonomic orders in the mammalian gut microbiome we interrogated (Figure 1B). Previously, LOG (Lonely Guy 1, CK activation enzyme)^22^ homologues were reported to be present across all major prokaryotic lineages except in species such as Escherichia coli and most obligate intracellular microbes with reduced genomes.^23^ Thus, the presence of CK-related genes in major gut microbial lineages is both plausible and consistent with our metagenomic analysis (Figure 1B and D). Likewise, we performed functional domain annotations for CK-related enzymes (activation and degradation), A- and B-type ARRs, and CK receptor genes identified in metagenomic data of the gut microbiomes (Figure 1B–D) retrieved from the GCMC database. We summarized domain counts, identities, and scores for SMART and Pfam, and included functional descriptions from eggNOG (protein names, GO terms, EC numbers, and KEGG pathways) to support meta-microbiome analysis (See Supplementary Tables 1–5). In this context, A- and B-type ARRs were characterized by prominent response regulator receiver domains and HSK domains, which are found in CK receptors and are integral to CK signaling. Activation enzymes consistently contained lysine decarboxylase domains (LOG family), while degradation enzymes showed FAD-binding and FAD-oxidase domains typical of CK dehydrogenases. These results support our metamicrobiome analysis, highlighting the distribution of CK-related gene families across microbial lineages, with comprehensive annotation details provided in Supplementary Tables 1–5.

**Figure 2.**
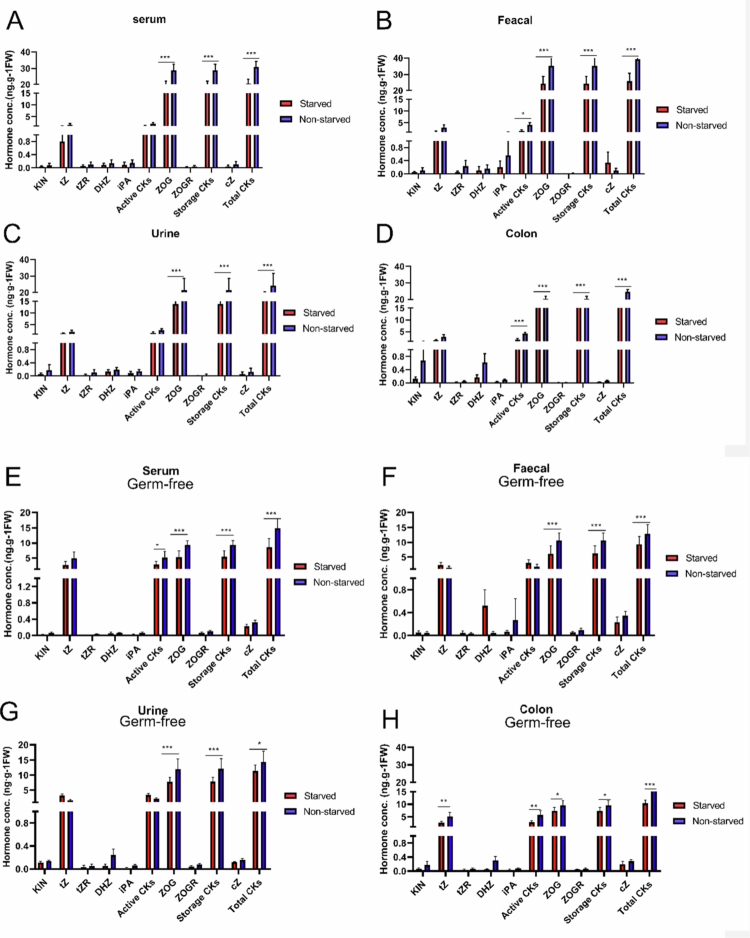
Quantitative analysis of cytokinin metabolites of normal and germ-free mice samples under starved and non-starved conditions. Cytokinin hormone concentrations in different biological matrices from starved and non-starved C57BL mice samples (A–D). Serum (A); fecal samples (B); urine (C); and colon (D). Cytokinin hormone concentrations in corresponding germ-free C57BLJ/6-mice under starved and non-starved conditions (E–H). Serum (E); fecal samples (F); urine (G); colon (H). Where KIN, kinetin; tZ, trans-zeatin; iZR, isopentenyladenosine riboside; DHZ, dihydrozeatin; iPA, isopentenyladenine; active CKs, active cytokinins; ZOG, zeatin-O-glucoside; ZOGR, zeatin-O-glucoside riboside; storage CKs, conjugated storage forms of cytokinins; cZ, cis-zeatin; total CKs, sum of all quantified cytokinin metabolites; FW, fresh weight. Data are presented as mean ± SEM (*n* = 4). Normality of the data was assessed prior to analysis and indicated deviation from normal distribution; therefore, statistical comparisons between starved and non-starved groups were performed using unpaired non-parametric Mann–Whitney tests. *p < 0.05, **p < 0.01, ***p < 0.001 indicate statistically significant differences between starved and non-starved C57BL mice groups or between starved and non-starved germ-free C57BLJ/6-mice groups.

Next, in order to investigate the dietary contribution, we conducted feeding and starvation experiments in mice. Our analysis revealed that total CK content in the sera of starved C57BL mice differed significantly from that of mice maintained under non-starved conditions (Figure 2A). Likewise, the concentration of storage CK forms was significantly higher in the sera of non-starved mice than in starved ones (Figure 2A). Further analysis of mouse colon tissues, fecal material, and urine samples under fed and starved conditions revealed a consistent pattern: CK levels, including both storage forms and total CK, were higher in the colon, feces, and urine of fed mice compared to the starved cohort (Figure 2B–D). These findings support the conclusion that dietary intake has a profound influence on the CK pool in mouse body tissues and fluids. Independently, CK profiling of the standard mouse diet confirmed the presence of various CK types (total CKs: 9.456 ± 1.083 ng·g^−1^, storage CKs: 7.214 ± 1.479 ng·g^−^^1^), albeit in minute quantities, indicating that diet is a contributing factor to the systemic CK pool. Statistical analysis for normality and is given in Supplementary doc, Tables 5 and 6A–G.

Lastly, the persistent presence of residual CK levels in starved mice suggests the existence of additional sources that maintain CKs in the serum (Figure 2B–D). Based on our microbiome and metagenomic analyses, we identified CK metabolic and signaling genes within the gut microbial communities of the studied mammalian species, including mice (Figure 1B), indicating a microbial capacity for CK production in the gut environment. We therefore hypothesize that the gut microbiome also contributes to maintaining serum CK levels. To test this, we analyzed CK levels in germ-free C57BL/6 mice under fed and starved conditions (Figure 2E–H). CK levels in all sample types—serum, feces, urine, and colon- were significantly lower in germ-free mice than in their conventionally raised counterparts (Figure 2A–D). This underscores the role of gut microbiomes in CK modulation, possibly via CK-related metabolic pathways. Notably, these observations align with previous work linking microbiota to the modulation of plant hormone-like indolic compounds such as auxins (indole-3-acetic acid: IAA and indole butyric acid: IBA),^24^ which act in coordination with CKs in plants.^25^ Despite growing evidence that plant hormones can regulate key mammalian processes,^16^ their role within the gut microbiome-food nexus remains in its infancy. With this study, we show that in tandem, CKs are microbiome-modulated metabolites in the C57BL mouse model (Figures 1B, 2E–H), marking the first step toward defining their role in this interface. A detailed statistical comparison of all these groups (Comparison between normal vs germ-free in Urine (starved and non-starved) and their data is given in the Supplementary doc file (Table 7–10).

While the fasting experiment revealed a significant reduction in cytokinin levels across serum and peripheral samples, the relatively short fasting duration (8 h) warrants careful interpretation. In mice, this timeframe is generally sufficient to induce a post-absorptive state and reduce active nutrient uptake, yet it may not completely eliminate residual dietary compounds from the gastrointestinal tract. Therefore, part of the observed decrease in cytokinin levels could reflect diminished intestinal absorption rather than a full depletion of diet-derived pools. Importantly, extending fasting beyond this period (e.g., 24 h) can introduce substantial physiological stress, including metabolic reprogramming and alterations in gut microbiome composition, which themselves may influence cytokinin dynamics and confound attribution to dietary sources alone. Moreover, our conclusions are supported by complementary evidence, including the direct detection of cytokinins in the diet and the markedly reduced cytokinin levels observed in germ-free mice, underscoring a combined dietary and microbiome contribution. Nonetheless, future studies employing longer fasting durations or controlled cytokinin-depleted diets will be valuable for more precisely disentangling the relative contributions of intestinal contents versus systemic cytokinin pools.

Regarding endogenous CK production capability, we investigated the evolutionary history of the TRIT1 gene. An unrooted phylogeny comprised of TRIT1 homologs from 319 representative species was constructed using RaxML. We see that tRNA-isopentenyltransferase has a much wider taxonomic distribution, encompassing eukaryotic and prokaryotic lineages and all lifeforms, including plants, animals, and microbes, including archaea (Supplementary Figure 1). Animal TRIT1/MOD5 comes from yeast and thus is present in one clade different from plant homologs (Supplementary Figure 1A). More intriguingly, the TRIT1 homolog is ubiquitously distributed among most of the bacterial lineages found in environmental samples. Our phylogenetic analysis indicates that the enzyme systems that produce specific types of CKs are part of the mammalian genome (Supplementary Figure 1A). While TRIT1 is instrumental in the synthesis of cZ type CKs, we still have other forms of CKs (kinetin, iP, tZ, ZOG, and ZOGR, etc.) in our analyzed mice sera under germ-free and diet-deprived states (Figure 2E–H). Analogous to its limited contribution to the production of CKs in plants, the activity of TRIT1 is not sufficient to be fully ascribed to the pool of CK derivatives that we found in our analysis (Figures 1A, 2A–H). We retrieved the gene expression profile of the TRIT1 gene in the human body and observed a ubiquitous yet moderate expression across various tissues, with the gastrointestinal tract, brain, cardiac, and skin tissues exhibiting the highest expression levels (Figure S1-B). We thus conclude that CKs are not solely acquired through dietary intake or microbial symbiosis, but rather an endogenous genetic repertoire encoding the necessary biosynthetic enzymes seems also to be in place. Future studies employing TRIT1 loss-of-function mouse models or CRISPR/Cas-mediated targeting will be instrumental in elucidating its role in mammalian CK biology.

## Conclusion

Together, our applied integrated metagenomics and metabolomics approaches support our understanding of the origin of CKs, transferred from the gut into the bloodstream, involving the gut-food nexus. Our functional experiments, which involved modulated feeding and starvation conditions in normal and germ-free mice, significantly affected the concentrations of CKs in various tissues and body fluids. Starving and germ-free mice still retained detectable CK levels, suggesting that the mice have an additional endogenous capability to produce CKs. CK presence in various body tissues and fluids indicates its potential physiological relevance in the mammalian system. The observed microbiome-mediated fluctuations in CK levels and the enrichment of CK-associated bacterial gene families underscore the need for targeted experiments to elucidate CK's functions in microbial signaling, quorum sensing, and gut microbiome homeostasis. Likewise, dietary modulations through CK spike in or CK deprivation in mice, along with their phenotyping for aging, oxidative stress, and healthy lifespan markers, will further delineate their role in longevity and pathophysiological complications. Species-level functional characterization of bacterial lineages associated with CK secretion can be tailored by fecal transplants with implications for mitigating inflammatory bowel diseases. While the role of CKs is well elucidated in plants, their importance in microbial signaling and mammalian physiological well-being will render CKs a global cue for cross-kingdom communication in biology.

## Materials and methods

### Mouse experiments and treatment conditions

Twelve male C57BL/6 mice were obtained from Charles River Laboratories and housed in a standard animal care facility under controlled conditions, including a 12-h light/dark cycle and an ambient temperature of 24 °C ± 2 °C. Animals had free access to tap water and standard laboratory chow throughout the study.

Animal experiments were conducted at the animal facility Plaisant Castel Romano, Rome, Italy. All procedures were carried out in accordance with the ARRIVE guidelines, the European Communities Council Directive 2010/63/EU, and the Italian Ministry of Health regulations (DL 26/2014), and were approved by the Italian Ministry of Health and the local Institutional Animal Care and Use Committee (IACUC) at the Istituto Superiore di Sanità (Rome, Italy; protocol no. 195/2021-PR). Experiments were performed in a blinded manner. Animals were monitored, treated, and euthanized throughout the study by trained facility personnel and veterinarians who were not directly involved in the experimental work.

Following a one-week acclimatization period, the mice were randomly assigned into two experimental groups, each consisting of four animals. The first group served as the non-fasting control, while the second group underwent an 8-h fasting protocol. Prior to the fasting intervention, and after 24 h in their respective conditions, baseline samples—including blood, urine, and stool—were collected from all animals to establish reference values. The fasting procedure was then initiated for Group 2, while Group 1 continued to receive food and water ad libitum. At the end of the fasting period, all animals were euthanized, and samples were collected from key organs and tissues, including the heart, liver, ileum (gut), leg muscle, kidney, and intestinal stool content. Blood was collected via the retro-orbital sinus under appropriate anesthesia. To prepare serum, blood samples were left undisturbed at room temperature for 15–30 min to allow clotting, followed by centrifugation at 4500 rpm for 10 min at 4 °C. The resulting supernatant (serum) was carefully collected and stored at −80 °C until analysis. All tissue samples were promptly frozen in liquid nitrogen, mechanically homogenized, and stored at −80 °C for cytokinin analysis.

### Meta-microbiome analysis for cytokinin-related gene families

Protein sequences implicated in CK signaling, metabolism, and transport (Table 1) were compiled in FASTA format, including representatives from the following functional categories: *cytokinin transporters, histidine phosphotransfer proteins, cytokinin inactivation enzymes, cytochrome P450 monooxygenases, cytokinin activation enzymes, cytokinin degradation enzymes, A-type ARRs, B-type ARRs, sensor histidine kinases,* and *IPT-class cytokinin production enzymes*. These sequences were used as queries for homology searches against the Global Microbial Gene Catalog (GMGC v1;.^26^ In brief, the GMGC is a comprehensive database derived from more than 13,000 curated metagenomes processed using a standardized computational pipeline. The catalog includes >2 billion open reading frames (ORFs), which were first clustered into 303 million unigenes based on 95% nucleotide identity, and subsequently grouped into 32 million protein families through distant homology clustering. This resource allows searching for sequence similarity across multiple biomes (human, pig, cat, dog, and mouse gut).

Sequence similarity searches were conducted using DIAMOND profile.^26^ Hits with an E-value ≤ 1 × 10^−3^ were retained as significant and subjected to downstream analysis. The sequences of these hits were compiled in FASTA format along with associated metadata such as sequence identifiers and alignment statistics. To annotate the taxonomic and ecological context of the identified homologs, a custom Python script was developed to query the GMGC API. This script retrieved taxonomic ranks and habitat metadata for each significant hit. The resulting enriched dataset was analyzed and visualized in R (v2023.09.1+494) using the ggplot2 package.

### Quantification of cytokinin metabolites in animal sera and body tissues

Cytokinins (CKs) were extracted and quantified following a refined protocol developed by Albacete et al.^27^ at Instituto Murciano de Investigación y Desarrollo Agrario y Alimentario: La Alberca, Murcia, ES. The method utilizes high-resolution mass spectrometry (HPLC-HRMS) for the sensitive and specific detection of a broad range of cytokinin types and derivatives in tissues. All solvents used throughout the procedure were of HPLC-MS grade, and deionized water was obtained from a Milli-Q system (18.2 MΩ·cm at 25 °C). Chemicals and reagents were used without further purification. A mixture of standard CKs, including trans-zeatin (Z), isopentenyladenine (iP), dihydrozeatin (DHZ), isopentenyladenine 9-glucoside (iP9G), trans-zeatin riboside (ZR), and trans-zeatin 9-glucoside (Z9G), was prepared in methanol: water (80:20, v/v) at a concentration of 0.4 µg/mL. Corresponding deuterated internal standards were prepared at the same concentration to enable precise quantification and correction for recovery. For extraction, fresh or lyophilized mammalian tissues or sera (minimum 75 mg fresh weight or 20 mg dry weight) were immediately flash-frozen in liquid nitrogen and stored at −80 °C until analysis. Tissues were ground to a fine powder using a ball mill or mortar and pestle under cold conditions. A precise amount of homogenized material was then suspended in an internal standard solution (10 ng/mL), and methanol: water (80:20, v/v) was added at 5 or 20 µL per mg of fresh or dry tissue, respectively. Samples were vortexed and incubated on an orbital shaker at 4 °C for 30 min.

Following extraction, samples were centrifuged at 20,000 × *g* for 15 min at 4 °C. The supernatant was carefully collected, and the pellet was re-extracted using the same procedure. Supernatants were combined and subjected to solid-phase extraction (SPE) using C18 cartridges (Chromafix, Macherey-Nagel), which had been pre-equilibrated with extraction solvent. The flow-through was collected, and solvents were evaporated under vacuum at 40 °C and 400 rpm for approximately three hours. Dried mammalian samples were resuspended in methanol: water (20:80, v/v), sonicated for 10 min in an ultrasonic bath to aid dissolution, and centrifuged again at 20,000 × *g* to remove particulates. The final extracts were transferred to 96-well plates along with calibration standards and stored at −80 °C until analysis. The animal sera (pig, human, mouse) were purchased from Thermo Scientific, Germany, while sera from cat and dog were obtained from Avantor, Germany, and lyophilized prior to analysis. The conventional and germ-free mice were obtained from Charles Rivers Laboratories.

Cytokinin quantification was performed using an ultra-high-performance liquid chromatography system (Accela U-HPLC, Thermo Fisher Scientific) coupled to an Ex-active Orbitrap mass spectrometer equipped with a heated electrospray ionization (HESI) interface. Chromatographic separation was achieved on an Accucore reversed-phase column (50 × 2.1 mm, 2.6 µm particle size). A gradient elution program with aqueous and organic mobile phases was used, optimized for CK separation. Samples were analyzed in negative ionization mode ([M-H]−), which was pre-tuned for a wide range of plant hormones. The MS acquisition included total ion chromatograms, from which compound-specific chromatograms were extracted using a 5-ppm mass tolerance window. For quantification of cytokinins, calibration curves were constructed for each analyzed component (1, 10, 50, and 100 µg l^−^^1^) and corrected for 10 µg l^−^^1^ deuterated internal standards. Recovery percentages ranged between 92% and 95%.

### Statistical analysis

Figure 1: Statistical analyses were performed using GraphPad Prism version 8.0 (GraphPad Software, San Diego, CA, USA). Data are presented as mean ± SEM of independent replicates. Comparisons of hormone concentrations across multiple species (tomato, pig, human, mouse, dog, and cat) were analyzed using one-way analysis of variance (ANOVA) followed by Tukey's multiple comparisons test to account for differences between groups. *p < 0.05 indicates statistically significant differences of cytokinin hormones in tomato compared with mammalian tissues.

Figure 2: All statistical analyses were performed using GraphPad Prism version 8.0 (GraphPad Software, San Diego, CA, USA). Data are expressed as mean ± SEM of biological replicates. Comparisons between starved and non-starved groups were conducted using an unpaired two-tailed Student's *t*-test for each cytokinin metabolite within the respective biological sample (serum, fecal, urine, and colon). *p < 0.05, **p < 0.01, ***p < 0.001 indicate statistically significant differences between starved and non-starved groups, both for conventionally grown and germ-free C57BL/6.

## Supplementary Material

Supplementary material Supp_table5_AHK_annotations.xlsx

Supplementary material Supp_table_3_ctokinin_activation_annotations - Kopie.xlsx

Supplementary material Supp_table_2_BRR_annotations.xlsx

Supplementary material SupplementaryMaterial.docx

Supplementary material Supp_table_4_ctokinin_degradation_annotations - Kopie.xlsx

Supp_table_1_ARR_annotations.xlsx Supp_table_1_ARR_annotations.xlsx

## Data Availability

All data and scripts used are made full available at DOI 10.5281/zenodo.18494785.
