# Supplementary material for: Diet and microbiome shape small-molecule cytokinin pools in mammals
Source: Gut Microbes. 2026 Jun 12;18(1):2679497. doi: 10.1080/19490976.2026.2679497 (PMC13274119; doi:10.1080/19490976.2026.2679497)
Supplement: Supplementary material — SupplementaryMaterial.docx [file KGMI_A_2679497_SM7584.docx]

**Domain Annotation for Metagenome candidates**

For the enzymes involved in cytokinin activation, degradation, and A-type and B-type ARRs, as well as the AHK receptors, which we identified in metagenomes, we extracted functional domain annotation summaries from the GMGC database, including SMART, PFAM, and eggNOG results. We summarized the number of detected domains, identities, and scoring information for SMART and Pfam. For eggnNOG annotations, we provide functional descriptions, including predicted protein names, GO terms, EC numbers, and KEGG pathway annotations. For the A-type and B-type ARRs transcription factors, the response regulator receiver domain was found to be most prominent, which have been reported to be a critical component of the cytokinin signaling pathway in plants (Supplementary Tables 1 and 2) [PMCID: PMC4012582]. Cytokinin activation enzyme hits consistently reported to contain a lysine decarboxylase domain and belong to the LOG family of proteins (Supplementary Table 3). For cytokinin degradation enzymes, FAD-binding and FAD-oxidase domains were found, which have been reported to be part of cytokinin dehydrogenase enzymes (Supplementary Table 4) [PMID: 34757471]. This further confirms and establishes the *in-silico* functionality of the identified enzymes. The domain annotation results for each enzyme class found in metagenomes are provided in Supplementary Tables 1-5.

**A**.


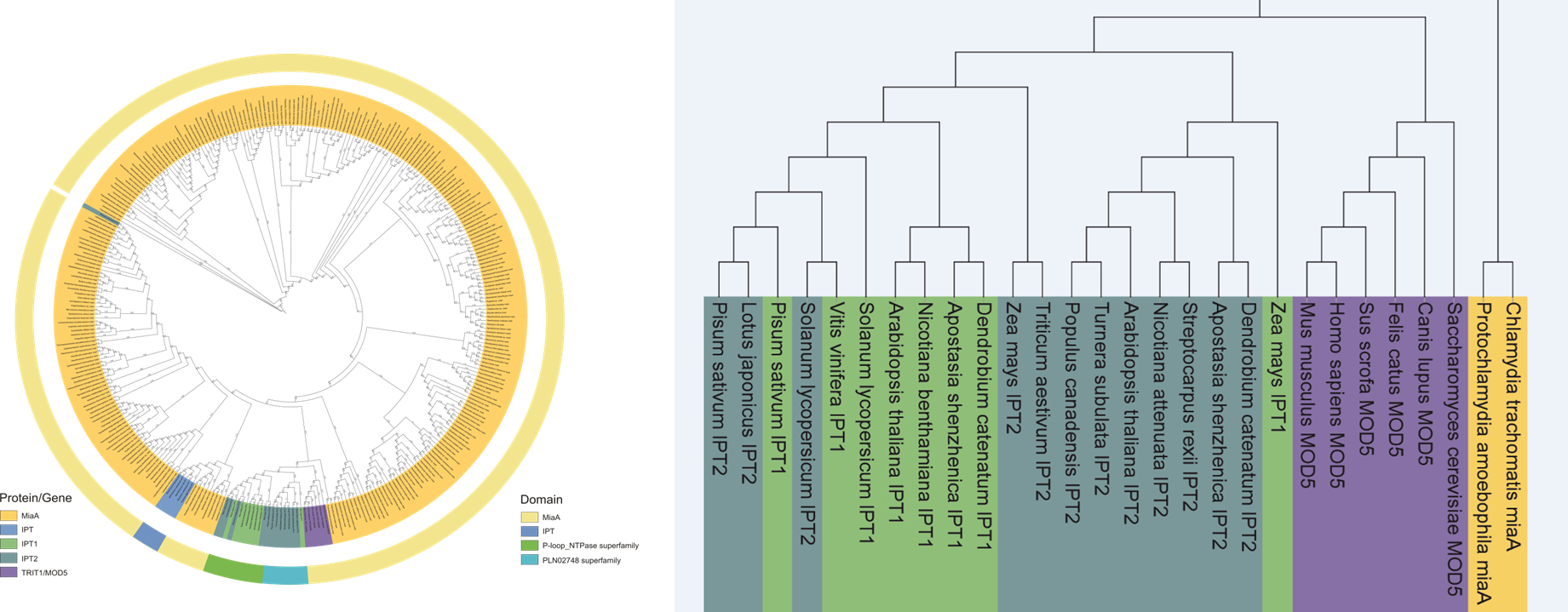


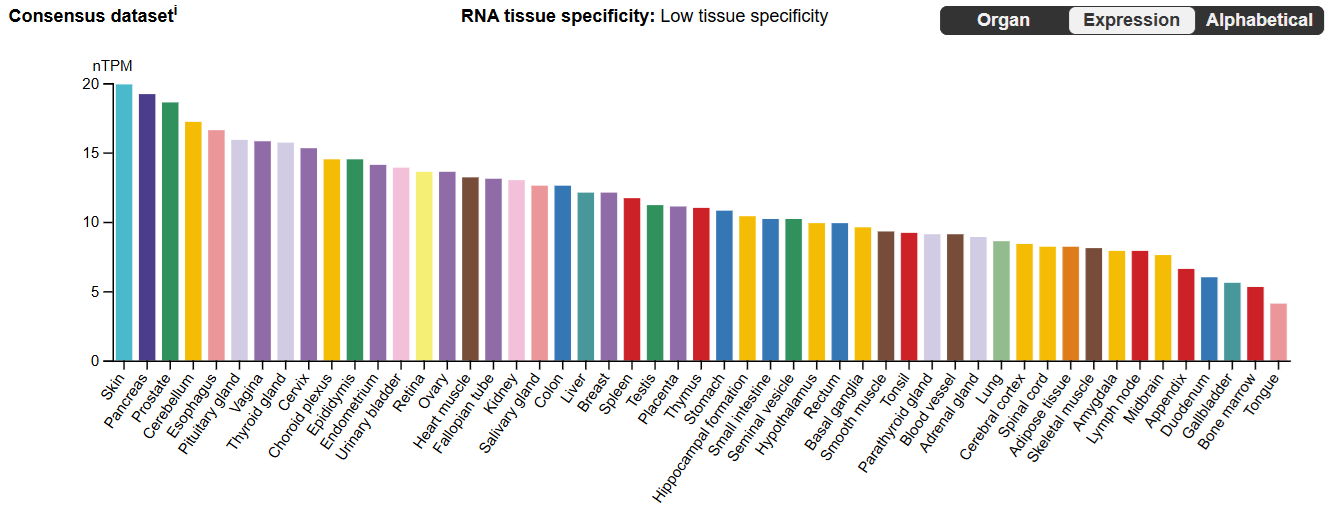


**B.**

Supplementary Figure 1: **(A) TRIT1-based phylogeny, domain-specific analysis, and expression values across multiple organisms**: A total of 319 protein sequences corresponding to Adenylate isopentenyltransferase and tRNA dimethylallyltransferase were retrieved from UniProt, including both eukaryotic and prokaryotic entries. Sequences were classified by gene name to distinguish proteins with similar nomenclature. Domain analysis was conducted using the NCBI batch conserved domain search, and only the major domain of each protein was considered for classification. Multiple sequence alignment was performed using Clustal Omega within the Geneious suite under default parameters. Phylogenetic analysis was carried out using RAxML in Geneious, employing the GAMMA BLOSUM62 protein model with the rapid hill-climbing algorithm and default settings. The resulting tree was further annotated and visualized using iTOL, and domain information was incorporated using CorelDRAW (SVG editor). (B) Organ-specific expression levels of the TRIT1 gene, quantified as normalized Transcripts Per Million (nTPM), were derived from RNA sequencing (RNA-seq) data obtained from the Human Protein Atlas (<https://www.proteinatlas.org/ENSG00000043514-TRIT1/tissue#rna_expression>).

**Supplementary** **Table 5: Normality assessment of cytokinin concentrations across different species**

| **Species** | **Shapiro–Wilk (W, p)** | **Anderson–Darling (p)** | **KS (p)** | **Conclusion** |
| --- | --- | --- | --- | --- |
| Tomato | 0.810, 0.0126 | 0.021 | 0.048 | Non-normal |
| Pig | 0.711, 0.0006 | 0.0004 | 0.0053 | Non-normal |
| Human | 0.713, 0.0007 | 0.0004 | 0.0073 | Non-normal |
| Mouse | 0.670, 0.0002 | <0.0001 | 0.0004 | Non-normal |
| Dog | 0.718, 0.0008 | 0.0005 | 0.0081 | Non-normal |
| Cat | 0.655, 0.0001 | <0.0001 | 0.0002 | Non-normal |

**W**, Shapiro–Wilk test statistic; **p**, p-value. Normality assessed using Shapiro–Wilk, Anderson–Darling (AD), and Kolmogorov–Smirnov (KS) tests. *p < 0.05 indicates deviation from normality

**Supplementary** **Table 6: Normality assessment of cytokinin concentrations across sample types and experimental conditions**

1. **Urine (Germ-free mice)**

| **Test** | **Starved (n=11)** | **Non-starved (n=11)** |
| --- | --- | --- |
| **Shapiro–Wilk (W, p)** | 0.771, 0.004 | 0.677, 0.0002 |
| **Anderson–Darling (A², p)** | 1.122, 0.0036 | 1.708, <0.0001 |
| **Kolmogorov–Smirnov (D, p)** | 0.311, 0.0038 | 0.348, 0.0006 |
| **D’Agostino–Pearson (K², p)** | 2.717, 0.257 | 3.299, 0.192 |
| **Conclusion** | Non-normal | Non-normal |

**W**, Shapiro–Wilk test statistic; **p**, p-value. Normality assessed using Shapiro–Wilk, Anderson–Darling (AD), and Kolmogorov–Smirnov (KS) tests. *p < 0.05 indicates deviation from normality.

**B. Urine (Normal mice)**

| **Test** | **Starved** | **Non-starved** |
| --- | --- | --- |
| **Shapiro–Wilk (W, p)** | 0.645, <0.0001 | 0.647, <0.0001 |
| **Anderson–Darling (A², p)** | 1.904, <0.0001 | 1.898, <0.0001 |
| **Kolmogorov–Smirnov (D, p)** | 0.381, <0.0001 | 0.378, <0.0001 |
| **D’Agostino–Pearson (K², p)** | 3.389, 0.184 | 3.387, 0.184 |
| **Conclusion** | Non-normal | Non-normal |

**W**, Shapiro–Wilk test statistic; **p**, p-value. Normality assessed using Shapiro–Wilk, Anderson–Darling (AD), and Kolmogorov–Smirnov (KS) tests. *p < 0.05 indicates deviation from normality.

**C. Serum (Germ-free mice)**

| **Test** | **Starved** | **Non-starved** |
| --- | --- | --- |
| **Shapiro–Wilk (W, p)** | 0.791, 0.0071 | 0.790, 0.0069 |
| **Anderson–Darling (A², p)** | 1.003, 0.0074 | 1.010, 0.0071 |
| **Kolmogorov–Smirnov (D, p)** | 0.304, 0.0055 | 0.308, 0.0044 |
| **D’Agostino–Pearson (K², p)** | 2.810, 0.245 | 2.750, 0.253 |
| **Conclusion** | Non-normal | Non-normal |

**W**, Shapiro–Wilk test statistic; **p**, p-value. Normality assessed using Shapiro–Wilk, Anderson–Darling (AD), and Kolmogorov–Smirnov (KS) tests. *p < 0.05 indicates deviation from normality.

**D. Serum (Normal mice)**

| **Test** | **Starved** | **Non-starved** |
| --- | --- | --- |
| **Shapiro–Wilk (W, p)** | 0.607, <0.0001 | 0.615, <0.0001 |
| **Anderson–Darling (A², p)** | 2.152, <0.0001 | 2.101, <0.0001 |
| **Kolmogorov–Smirnov (D, p)** | 0.418, <0.0001 | 0.410, <0.0001 |
| **D’Agostino–Pearson (K², p)** | 3.481, 0.175 | 3.467, 0.177 |
| **Conclusion** | Non-normal | Non-normal |

**W**, Shapiro–Wilk test statistic; **p**, p-value. Normality assessed using Shapiro–Wilk, Anderson–Darling (AD), and Kolmogorov–Smirnov (KS) tests. *p < 0.05 indicates deviation from normality.

**E. Colon (Germ-free mice)**

| **Test** | **Starved** | **Non-starved** |
| --- | --- | --- |
| **Shapiro–Wilk (W, p)** | 0.760, 0.0028 | 0.793, 0.0076 |
| **Anderson–Darling (A², p)** | 1.185, 0.0024 | 0.986, 0.0082 |
| **Kolmogorov–Smirnov (D, p)** | 0.301, 0.0063 | 0.313, 0.0035 |
| **D’Agostino–Pearson (K², p)** | 2.863, 0.239 | 2.880, 0.237 |
| **Conclusion** | Non-normal | Non-normal |

**W**, Shapiro–Wilk test statistic; **p**, p-value. Normality assessed using Shapiro–Wilk, Anderson–Darling (AD), and Kolmogorov–Smirnov (KS) tests. *p < 0.05 indicates deviation from normality.

**F. Fecal (Germ-free mice)**

| **Test** | **Starved** | **Non-starved** |
| --- | --- | --- |
| **Shapiro–Wilk (W, p)** | 0.789, 0.0068 | 0.680, 0.0003 |
| **Anderson–Darling (A², p)** | 1.000, 0.0076 | 1.689, 0.0001 |
| **Kolmogorov–Smirnov (D, p)** | 0.277, 0.0181 | 0.354, 0.0004 |
| **D’Agostino–Pearson (K², p)** | 3.213, 0.201 | 3.326, 0.190 |
| **Conclusion** | Non-normal | Non-normal |

**W**, Shapiro–Wilk test statistic; **p**, p-value. Normality assessed using Shapiro–Wilk, Anderson–Darling (AD), and Kolmogorov–Smirnov (KS) tests. *p < 0.05 indicates deviation from normality.

**G. Fecal (Normal mice**)

| **Test** | **Starved** | **Non-starved** |
| --- | --- | --- |
| **Shapiro–Wilk (W, p)** | 0.615, <0.0001 | 0.644, <0.0001 |
| **Anderson–Darling (A², p)** | 2.099, <0.0001 | 1.915, <0.0001 |
| **Kolmogorov–Smirnov (D, p)** | 0.412, <0.0001 | 0.382, <0.0001 |
| **D’Agostino–Pearson (K², p)** | 3.472, 0.176 | 3.401, 0.183 |
| **Conclusion** | Non-normal | Non-normal |

**W**, Shapiro–Wilk test statistic; **p**, p-value. Normality assessed using Shapiro–Wilk, Anderson–Darling (AD), and Kolmogorov–Smirnov (KS) tests. *p < 0.05 indicates deviation from normality.

**Comparison between normal vs germ-free in Urine (starved and non-starved)**

No statistically significant differences were observed for most cytokinin metabolites between conventional and germ-free mice. While some metabolites (e.g., *tZ, Active CKs, ZOG, Storage CKs*) showed trends toward differences, these did not reach statistical significance after appropriate non-parametric testing.
Our finding showed that Urinary cytokinin levels appear less sensitive to microbiota status, particularly under the studied conditions.

**Supplementary** **Table 7: A. Compare Normal vs germ-free for starved in Urine**

|  | Significant? | P value | Mean of Normal starved | Mean of Germ free starved |
| --- | --- | --- | --- | --- |
| KIN | No | 0.946647 | 0.06749 | 0.1107 |
| tZ | No | 0.011357 | 1.511 | 3.206 |
| tZR | No | 0.997086 | 0.02831 | 0.03067 |
| DHZ | No | 0.849377 | 0.1779 | 0.05533 |
| iPA | No | 0.852350 | 0.1374 | 0.01733 |
| Active CKs | No | 0.024188 | 1.922 | 3.419 |
| ZOG | No | 0.008842 | 9.546 | 7.789 |
| ZOGR | No | 0.962374 | 0.01090 | 0.04133 |
| Storage CKs | No | 0.009992 | 9.557 | 7.830 |
| cZ | No | 0.992552 | 0.1237 | 0.1177 |
| Total CKs | No | 0.861878 | 11.48 | 11.37 |

**B. Compare Normal vs germ free non-starved Urine**

|  | Significant? | P value | Mean of Germ free non starved urine | Mean of normal non starved urine |
| --- | --- | --- | --- | --- |
| KIN | No | 0.896989 | 0.1400 | 0.3253 |
| tZ | No | 0.973036 | 1.625 | 1.673 |
| tZR | No | 0.992086 | 0.05467 | 0.04047 |
| DHZ | No | 0.996545 | 0.2423 | 0.2361 |
| iPA | No | 0.950083 | 0.05800 | 0.1476 |
| Active CKs | No | 0.832253 | 2.120 | 2.423 |
| ZOG | No | 0.017847 | 11.98 | 15.48 |
| ZOGR | No | 0.983702 | 0.07633 | 0.04710 |
| Storage CKs | No | 0.018742 | 12.06 | 15.53 |
| cZ | No | 0.959475 | 0.1570 | 0.2297 |
| Total CKs | No | 0.014575 | 14.34 | 17.95 |

**Comparison between normal vs germ-free in Serum (starved and non-starved)**

Significant differences were observed for ZOG, Storage CKs, and Total CKs were consistently and significantly reduced in germ-free mice (*p < 0.000001*). Additional differences were noted for *tZ* and *Active CKs* in the non-starved condition.
Serum cytokinin profiles show robust microbiota-dependent differences, supporting a systemic contribution of the microbiota to cytokinin levels.

**Table 8: A. Compare normal vs germ free starved serum**

|  |  | Significant? | P value | Mean of normal starved serum | Mean of germ-free starved serum |
| --- | --- | --- | --- | --- | --- |
| KIN |  | No | 0.997022 | 0.02459 | 0.02150 |
| tZ |  | No | 0.013171 | 0.6529 | 2.756 |
| tZR |  | No | 0.979521 | 0.03377 | 0.01250 |
| DHZ |  | No | 0.923751 | 0.1360 | 0.05675 |
| iPA |  | No | 0.869546 | 0.1611 | 0.02500 |
| Active CKs |  | No | 0.027350 | 1.008 | 2.871 |
| ZOG |  | Yes | <0.000001 | 19.47 | 5.333 |
| ZOGR |  | No | 0.945572 | 0.01244 | 0.06900 |
| Storage CKs |  | Yes | <0.000001 | 19.48 | 5.403 |
| cZ |  | No | 0.796098 | 0.008875 | 0.2230 |
| Total CKs |  | Yes | <0.000001 | 20.49 | 8.496 |

**B. Normal vs germ-free non starved serum**

|  | Significant? | P value | Mean of normal non starved serum | Mean of germ-free non starved serum |
| --- | --- | --- | --- | --- |
| KIN | No | 0.990302 | 0.07612 | 0.06125 |
| tZ | Yes | 0.002892 | 1.164 | 4.934 |
| tZR | No | 0.989276 | 0.04794 | 0.03150 |
| DHZ | No | 0.900942 | 0.2222 | 0.07000 |
| iPA | No | 0.901659 | 0.2206 | 0.06950 |
| Active CKs | No | 0.006342 | 1.731 | 5.166 |
| ZOG | Yes | <0.000001 | 30.43 | 9.283 |
| ZOGR | No | 0.941639 | 0.01721 | 0.1068 |
| Storage CKs | Yes | <0.000001 | 30.44 | 9.390 |
| cZ | No | 0.817767 | 0.03989 | 0.3218 |
| Total CKs | Yes | <0.000001 | 32.17 | 14.88 |

**Comparison between normal vs germ-free in colon (starved and non-starved)**

A significant reduction in germ-free mice were observed for ZOG, Storage CKs, and Total CKs (*p < 0.000001*). while other metabolites showed no significant differences. Colon tissue demonstrates strong microbiota-associated modulation, consistent with its direct interaction with gut microbes.

**Table 9: A. Normal vs germ free starved colon**

|  | Significant? | P value | Mean of normal starved colon | Mean of germ-free starved colon |
| --- | --- | --- | --- | --- |
| KIN | No | 0.923326 | 0.1215 | 0.06475 |
| tZ | No | 0.029414 | 1.323 | 2.632 |
| tZR | No | 0.973868 | 0.01993 | 0.03925 |
| DHZ | No | 0.837157 | 0.1678 | 0.04650 |
| iPA | No | 0.985261 | 0.02835 | 0.03925 |
| Active CKs | No | 0.052434 | 1.661 | 2.822 |
| ZOG | Yes | <0.000001 | 16.10 | 7.300 |
| ZOGR | No | 0.956734 | 0.01350 | 0.04550 |
| Storage CKs | Yes | <0.000001 | 16.11 | 7.346 |
| cZ | No | 0.765752 | 0.02144 | 0.1973 |
| Total CKs | Yes | <0.000001 | 17.77 | 10.36 |

**B. Normal vs germ-free non starved colon**

|  | Significant? | P value | Mean of normal non-starved colon | Mean of germ-free non starved colon |
| --- | --- | --- | --- | --- |
| KIN | No | 0.567138 | 0.6769 | 0.1710 |
| tZ | No | 0.015207 | 2.903 | 5.096 |
| tZR | No | 0.977487 | 0.04258 | 0.06750 |
| DHZ | No | 0.726532 | 0.6187 | 0.3098 |
| iPA | No | 0.985336 | 0.09123 | 0.07500 |
| Active CKs | No | 0.119771 | 4.333 | 5.719 |
| ZOG | Yes | <0.000001 | 20.24 | 9.515 |
| ZOGR | No | 0.948559 | 0.01353 | 0.07050 |
| Storage CKs | Yes | <0.000001 | 20.25 | 9.585 |
| cZ | No | 0.805369 | 0.06738 | 0.2850 |
| Total CKs | Yes | <0.000001 | 24.58 | 15.59 |

**Comparison between normal vs germ-free in faeces (starved and non-starved)**

ZOG, Storage CKs, and Total CKs were significantly reduced in germ-free mice (*p < 0.000001*) while other metabolites did not show significant differences. Fecal cytokinin profiles provide direct evidence of microbial contribution**,** as expected given the microbial origin of fecal metabolites.

**Table 10: A. Normal vs germ-free starved faecal**

|  | Significant? | P value | Mean of normal starved faecal | Mean of germ-free starved faecal |
| --- | --- | --- | --- | --- |
| KIN | No | 0.989142 | 0.03643 | 0.05150 |
| tZ | No | 0.309287 | 1.195 | 2.325 |
| tZR | No | 0.993967 | 0.05037 | 0.04200 |
| DHZ | No | 0.772086 | 0.1992 | 0.5200 |
| iPA | No | 0.783643 | 0.3729 | 0.06875 |
| Active CKs | No | 0.299664 | 1.854 | 3.007 |
| ZOG | Yes | <0.000001 | 27.01 | 6.088 |
| ZOGR | No | 0.961868 | 0.001811 | 0.05475 |
| Storage CKs | Yes | <0.000001 | 27.01 | 6.143 |
| cZ | No | 0.886214 | 0.07379 | 0.2323 |
| Total CKs | Yes | <0.000001 | 28.86 | 9.382 |

**B. Normal vs germ-free non starved fecal**

|  | Significant? | P value | Mean of normal non starved fecal | Mean of germ free non starved fecal |
| --- | --- | --- | --- | --- |
| KIN | No | 0.992442 | 0.03549 | 0.04875 |
| tZ | No | 0.613530 | 2.131 | 1.423 |
| tZR | No | 0.961357 | 0.1018 | 0.03400 |
| DHZ | No | 0.875577 | 0.2620 | 0.04275 |
| iPA | No | 0.565654 | 1.076 | 0.2708 |
| Active CKs | No | 0.204755 | 3.606 | 1.820 |
| ZOG | Yes | <0.000001 | 37.04 | 10.66 |
| ZOGR | No | 0.952910 | 0.01382 | 0.09650 |
| Storage CKs | Yes | <0.000001 | 37.06 | 10.75 |
| cZ | No | 0.894508 | 0.1586 | 0.3443 |
| Total CKs | Yes | <0.000001 | 40.66 | 12.92 |
